# The evolution of non-communicable diseases policies in post-apartheid South Africa

**DOI:** 10.1186/s12889-018-5832-8

**Published:** 2018-08-15

**Authors:** Catherine Ndinda, Tidings P. Ndhlovu, Pamela Juma, Gershim Asiki, Catherine Kyobutungi

**Affiliations:** 1 Human Science Research Council, Pretoria, South Africa; 2 Development Studies, University of South Africa, Pretoria, South Africa; 3 Manchester Metropolitan University, Manchester, UK; 4 Graduate School of Business Leadership, University of South Africa, Pretoria, South Africa; 5 African Population Health Research Centre, Nairobi, Kenya

**Keywords:** Non-communicable diseases, Multi-sectoral approach, Policy analysis, Key informants, South Africa

## Abstract

**Background:**

Redressing structural inequality within the South African society in the post-apartheid era became the central focus of the democratic government. Policies on social and economic transformation were guided by the government’s blueprint, the Reconstruction and Development Programme. The purpose of this paper is to trace the evolution of non-communicable disease (NCD) policies in South Africa and the extent to which the multi-sectoral approach was utilised, while explicating the underlying rationale for “best buy” interventions adopted to reduce and control NCDs in South Africa. The paper critically engages with the political and ideological factors that influenced design of particular NCD policies.

**Methods:**

Through a case study design, policies targeting specific NCD risk factors (tobacco smoking, unhealthy diets, harmful use of alcohol and physical inactivity) were assessed. This involved reviewing documents and interviewing 44 key informants (2014–2016) from the health and non-health sectors. Thematic analysis was used to draw out the key themes that emerged from the key informant interviews and the documents reviewed.

**Results:**

South Africa had comprehensive policies covering all the major NCD risk factors starting from the early 1990’s, long before the global drive to tackle NCDs. The plethora of NCD policies is attributable to the political climate in post-apartheid South Africa that set a different trajectory for the state that was mandated to tackle entrenched inequalities. However, there has been an increase in prevalence of NCD risk factors within the general population. About 60% of women and 30% of men are overweight or obese. While a multi-sectoral approach is part of public policy discourse, its application in the implementation of NCD policies and programmes is a challenge.

**Conclusions:**

NCD prevalence remains high in South Africa. There is need to adopt the multi-sectoral approach in the implementation of NCD policies and programmes.

## Background

Rising mortality rates from non-communicable diseases (NCDs) globally present challenges to policy-makers. According to the WHO, NCDs result from a combination of genetic, physiological, environmental, and behavioural factors [1]. Deaths from NCDs are predicted to rise to 52 million by 2030, 80% of these will be in low- and middle-income countries. This calls for an urgent evaluation of policies that are designed to fight NCDs [2–6]. It is notable that there has been some progress and political commitment in South Africa in tackling HIV/AIDS and tuberculosis, and legislation for successfully reducing tobacco consumption, fatty acids, salt and sugar, as well as curbing advertisements of unhealthy food [7]. Notwithstanding this, 2 out of 5 deaths are attributable to NCDs. Limited resources and inadequate infrastructure in the health sector have exacerbated the situation [2, 4–6, 8–10].

The prevention of premature deaths requires an understanding of the primary NCD risk factors - unhealthy diets, tobacco smoking, physical inactivity and alcohol abuse. Analyses must explore not only how these risk factors account for the four main NCDs - cancer, diabetes, cardiovascular diseases and chronic respiratory diseases [5, 6] as major causes of mortality worldwide - but also how they dovetail with a multi-sectoral approach in South Africa.

An analysis of (NCD) prevention policies in Africa (ANPPA) (2013–2016) was done in five African countries (Kenya, Malawi, Cameroon, Nigeria and South Africa). The South African case study sought to explore the extent to which multi-sectoral action is used in the formulation and implementation of policies that are related to the four NCD risk factors [7]. The study also sought to establish the extent to which the WHO “best buy” [11] interventions were included in the NCD policies and programmes. The WHO describes “best buys” as “interventions that have significant public health impact and are highly cost-effective, inexpensive, and feasible to implement” [11]. The purpose of this paper is to trace and understand the evolution of NCD policies in South Africa since 1994. This involves the exploration of the policy context and the implications for applying a multi-sectoral action (MSA). The key objective is to explicate the underlying rationale for the way NCD prevention and control has been tackled in post-apartheid South Africa.

## Methods

The study employed a case study design [12]. A review of literature and policies that target NCD risk factors (tobacco use, unhealthy diet, physical inactivity and the harmful use of alcohol) was conducted. Two primary sources of data were utilised: (1) a desk review of relevant documents and (2) key informant interviews with those who either participated or had a keen interest in the policy process. This was part of ANPPA study that was coordinated by the African Population and Health Research Centre [13]. The study received Ethics approval from the Human Science Research Council Ethics Committee (REC 2/19/02/2014) and data collection took place from June 2014 to January 2016.

### Document reviews

The research team conducted document review to capture the policy context and content and identify existing policies and gaps therein. The research used Ebscohost web to access NCD policy documents focusing on four key risk factors (unhealthy diets, physical inactivity, tobacco smoking and harmful use of alcohol). This consisted of published and grey literature that included annual and strategic departmental reports, guidelines and programme materials. Also included were unpublished dissertations and conference papers. During the interviews with key informants, more documents that were not in the public domain were retrieved. Data extracted from the documents included identification of years in which relevant policy changes had occurred and the events leading up to those decisions.

### Key informant interviews

Purposive sampling and snowball sampling techniques were used to select the key informants. Following the Health-In-All-Policies model, a broad segment of sectors such as health, education and finance were identified for inclusion. This was followed by the identification of appropriate individuals within those sectors and institutions that purposively included both government and non-governmental actors. Attending a workshop convened by the South African Non-Communicable Diseases (SANCD) Alliance assisted in identifying most of the study participants [7]. Individuals recruited included senior decision makers in the selected sectors such as departmental or divisional heads or programme managers; heads of non-governmental organizations involved in NCD prevention programmes or projects; and heads of private sector institutions or departments and programmes within those institutions involved in NCD prevention. The identified informants helped the researchers to identify other key informants. Participants were contacted through telephone and email. Once they agreed to participate, scheduled interviews were arranged with informants and copies of the study information sheet and outline of the respective interviews were sent to them.

When the interviews began, most of the study participants referred to their participation in the formulation of the salt reduction regulations [7]. To ensure that participants also spoke about other policies (alcohol control, tobacco and physical activity), the research team changed tactic and at the beginning of each interview asked about involvement in the formulation process. Given that some of the policies were formulated in the 1990s, participants could speak about the policies they were more familiar with. Participants that were involved in advocacy confidently described the formulation process of policies such as tobacco control, and programmes on substance abuse, as well as physical activity. The gaps in recalling circumstances surrounding the formulation of policies such as those concerning substance abuse and physical activity were filled through the review of departmental documents and reports.

The interview guide included general questions on the policy context, process, sectors involved and barriers to policy formulation, and the application of multisectoral action (MSA) in policy design and implementation. This was designed to explore the formulation and implementation of policies that target NCD risk factors in South Africa.

The key informant interviews were electronically recorded, but in cases where individuals declined being recorded, the study team took notes [7]. The interviews were conducted at mutually agreed times and at venues that were free from distractions. The interviewers explained the purpose of the study, risks and benefits of participating, the right to withdraw at any time without penalty, and confidentiality, while participants provided verbal or written documentation of consent to participate.

Recorded interviews were transcribed, edited to remove typographical and grammatical errors and real names of study participants, and were saved with identification codes on password-protected servers. In line with ethical standards and to ensure anonymity, the study participants were identified by numbers 1–44. Transcripts were uploaded into the qualitative data management software NVivo. Guided by the key research questions, thematic analysis [7, 14] was used to code both documents and transcripts, and results were reported thematically in terms of how participants understood the evolution of NCD control policies in South Africa.

## Results

**Fig. 1:**
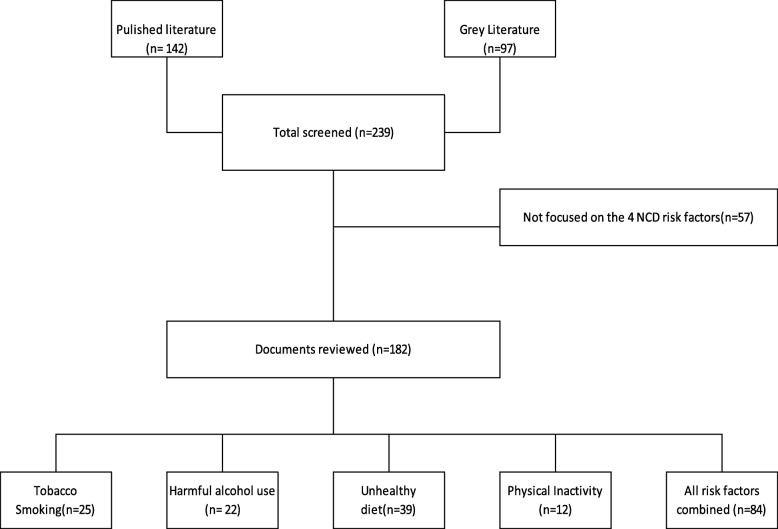
Documents screened and reviewed

As shown in Fig. 1, a total of 239 documents were retrieved for screening (142 published and 97 grey literature), and 57 were excluded because they were not relevant to the four NCD risk factors; thus 182 documents were reviewed (Fig. 1). The documents reviewed included Acts and laws, regulations, development policies, White papers, strategic plans, guidelines and government directives, reviews and case studies of multi-sectoral action with regards to policy formulation and implementation at the national level. Examples of policy documents included: departmental website materials such as policy documents, strategic plans, program plans, guidelines, protocols, media releases; speeches by politicians; workshop reports and drafts of policy statements; academic journal articles; and reports of relevant non-governmental organizations on NCD programs.

In total, 44 study participants were interviewed. As shown in Tables 1, 26 out of the 44 study participants were drawn from the health sector (health research institutions, health departments at universities, professional health associations and non-governmental organizations (NGOs) involved in advocacy for the prevention and control of NCDs). The rest were spread across different sectors such as finance, agriculture, social development, the private sector and industry. The section that follows reports the results in terms of the policy context, the NCD policies passed, the challenges of implementing NCD policies and the application of the multi-sectoral approach in NCD policies in South Africa.

**Table 1:** Study participants

Organisation	Number
Professional associations	4
Universities & research institutions	8
Non-governmental organisations	7
Health financiers	1
Departments of health - national, provincial & local	5
Other government departments	12
Industry	4
Media	2
Consulting firms	1
Total	44

### Policy context

#### Post-apartheid period

Having emerged from the context of institutionalised racial discrimination, segregation and inequality of access to healthcare resources, questions of equity were paramount in the minds of the majority of the African population in the immediate post-apartheid South Africa. The national discourse concerning health priorities was dominated by debates on equity and redistribution.

The ANC government in 1994 had a mission to eradicate the structural inequalities of the apartheid system and build “a democratic non-racial and non-sexist [society in the] future” ([15], pp. 4–5). Central to debates at the 1994 Bloemfontein Conference, 1997 Mafikeng Conference, and subsequent party conferences, were concerns about access to services, equitable redistribution of resources, and devolvement of power to the people, encapsulated in the dictum, *Amandla Awethu (*power to the people). The government was committed to a different health equity trajectory and the allocation of limited resources to the poorest and majority of the population [15–17].

NCDs did not feature prominently in the initial post-apartheid years but there were concerns about how to achieve health equity, redistribute resources to ensure wider access for the masses and how illnesses and diseases that affected the majority of people (especially in rural areas) were to be tackled. The ANC prioritised HIV and AIDS, which, by 1997, had reached pandemic proportions. The period, between 1994 to 2004, was devoted to tackling HIV/AIDS, and providing anti-retroviral treatment to people living with HIV and AIDS [7, 18].

**Fig. 2:**
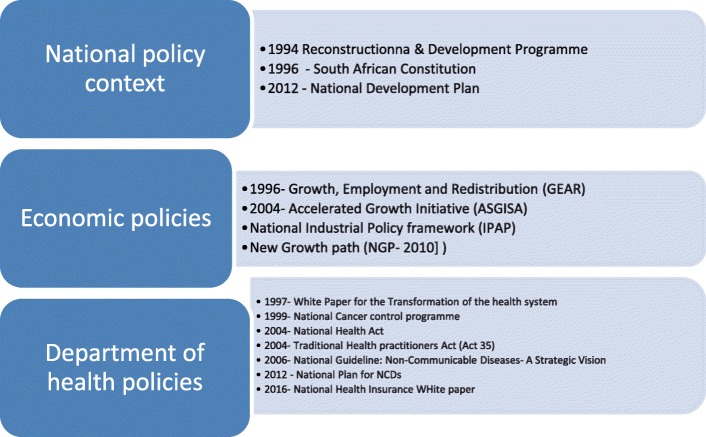
Policy context of non-communicable diseases

However, from 2009 the government increasingly focused on growing problems associated with NCDs. While there appeared to be a set-back in health policies with introduction of the Growth and Employment Policy (GEAR) (1996) that liberalised the economy and opened it up to international competition and cheap processed foods [19], the Department of Health (DOH) was emboldened by the post-apartheid Constitution (1996). There was also pressure for inclusive national development that enabled it to formulate disease-specific policies and guidelines to ensure the control and prevention of NCDs [7, 20]. As shown in Fig. 2, policies that evolved from 1994 to 2016 were influenced by the transformative agenda of tackling inequalities and, latterly, the burden of NCDs.

The NCD Directorate in the Department of Health was created in the immediate post-apartheid period (1996) but government rhetoric and actions initially focused on the NCD risk factors that were of particular concern to the general public (substance abuse – tobacco smoking, drugs, and alcohol). Although the Tobacco Control Act was enacted in 1993, the regulations and subsequent amendments were finalised in the post (1994)-apartheid period.

Between 1994 and 2015, the DOH developed more than 40 policies (guidelines, programmes, regulations and legislations), all with the aim of preventing, controlling and managing NCDs [7]. The key policies are summarised in Table 2 and these focus on the key risk factors and the “best buy” interventions. Various political events shaped the approach towards NCDs policy development.

**Table 2:** Summary of Policies targeting NCD Risk Factors

Risk Factors targeted	Specific policies
Tobacco smoking	1993-Tobacco Products Control Act 21
	1994-Tobacco Products Control Regulations
	1999-Tobacco Products Control Amendment Act 23
	2000-Tobacco Products Control Amendment
	2007-Tobacco Products Control Amendment Act 25
	2008-Tobacco Products Control Amendment Act 28
	2011-Tobacco Products Control Amendment- regulations
Alcohol Abuse	1989-Liquor Products Act 60
	2003-National Liquor Act 59
	2004-National Liquor Regulations
	2008-Western Cape Liquor Act; 2013 – Gauteng Liquor Act
	2013-National Drug Masterplan (2013–2017)
Unhealthy diets	2009-National School Nutrition policy
	2011-Regulations relating to trans-fat in foodstuffs
	2013-Food and Nutrition Security Policy by Department of Agriculture
	2015-Strategy for Tackling Obesity
	2013-Salt Reduction Regulations
Physical inactivity	1996-Schools Act 84
	1998-National Sports and Recreation Act
	2011-Promotion of Physical Activity in Older Persons
	2012–2016-National Strategic Plan for NCDs

#### Political will

In the 2009 national elections, Jacob Zuma became president and appointed Dr. Aaron Motsoaledi, a physician by training, as Minister for Health. Although HIV/ AIDS remained a key priority of government, the recognition that people on antiretroviral therapy (ARV) therapy were living long enough to contract NCDs prompted the government to focus on the co-morbidities of communicable and non-communicable diseases. Dr. Motsoaledi tackled the prevention and control of NCDs (alongside HIV and AIDS) as part and parcel of an overall strategy for reducing the disease burden in South Africa [20–22].

In 2010, the Department of Health’s (DOH) strategic document “Outcome 2” pronounced the country’s health targets for achieving vision 2030 [23]. The emphasis on ensuring a healthy life for all by the year 2030 was articulated through six objectives, two of which included achieving (i) “a significant shift in equity, efficiency and quality of health” and (ii) “significantly reduced determinants of disease and adverse ecological factors” ([24], p. 1). The focus on NCDs in Outcome 2, was reflected in the National Development Plan (NDP), popularly known as Vision 2030 [23]. In line with health equity goals, the government has since 2010, included the control and prevention of NCDs among its priorities.

In 2011, the DOH had already issued the declaration on the prevention and control of NCDs that, among others, committed the country to further develop and implement policies, strategies and surveillance systems for NCDs [7, 20]. Despite the existence of NCD policies since 1994, it was however not until 2012 that a comprehensive National Health Strategic Plan focusing on all NCDs was developed [25]. The Plan included mental health that had not been included in the WHO “Best buys”, but one that is a critical NCD in the South African context. The country went further to specify targets and dates for NCD reduction.

A motion on unhealthy lifestyles tabled at the 2012 ANC conference indicated that there should be a break from apartheid health policies: “The government should fast-track legislation and regulations to deal with the four risk factors, including the creation of a healthcare commission whose function is specifically to deal with the said risk factors” ([26], p. 77). The conference resolved that: “The ANC and government must embark on activities to promote healthy lifestyles through the mobilization of individuals and communities to engage in physical activities, good dietary practices and reduction of harmful use of alcohol, tobacco and control of substance abuse” ([26], p. 77). These concerns, coming 18 years after the ANC rose to office (2012), represented the clearest articulation of the government’s position on NCDs.

In the 2016 budget speech, the Minister for Health highlighted NCDs as of particular concern in South Africa. Subsequent policies and ministerial proclamations would be geared towards charting a new path that would ensure the prevention and control of NCDs, bridge the gap of health inequalities and inequities, and address the risk of NCDs by 2020.

#### The burden of NCDs

Indeed, in order to tackle premature deaths from NCDs, the government set out to reduce the mortality rate by 25% in 2020 [27]. Estimates by the DOH attributed 49% of deaths in the country to NCDs [22]. It was also established that people who lived with HIV and AIDS were vulnerable to NCDs such as cancer, heart disease, mental disorder, and diabetes, among others [4–6, 8, 9, 25]. In addition, malnutrition, low birth-weight were found to paradoxically predispose individuals to obesity, high blood pressure, heart disease and diabetes in adult life. These risk factors affected both mothers and children [4–6, 25, 27–29].

It is against this background that Dr. Motsoaledi, in his budget vote for health (2016), identified four epidemics (HIV and AIDS, maternal and child mortality, injuries and violence and NCDs), that he described as “the four highways [through which] South Africans are marching to their graves” ([21], p. 2). Recognition of the dangers of these “four colliding epidemics” ([21], p. 4) led to a national discourse on NCDs, and subsequently the adoption of a multi-sectoral approach to tackle the epidemic in line with the new path of equity.

In addition to the policies, the Strategic Plan for NCDs (2013–2017) and the WHO’s 2016–2020 country strategy provided a framework for reducing morbidity and mortality from non-communicable diseases [6, 25].

### The NCD policies

#### Policy on alcohol and substance abuse

The growing concerns about the impact of NCDs, especially alcohol and substance abuse, informed the development of the policy on alcohol control. In the light of ANC Conference resolutions and subsequent legislation from the 1990s to 2003, the government pledged to lead campaigns against substance abuse, including supporting programmes of rehabilitating and assisting addicts to reintegrate into society. An Inter-Ministerial Committee (IMC) was set up in 2010 to direct policies for this purpose [7]. The proposed policies involved zero tolerance with regards to “drink and driving”, taxation, normalisation of the previously illegal drinking houses (during apartheid) *Sheebens*, “zoning” these houses in appropriate places and regulating drinking hours, raising the legal age for drinking from 18 to 21 and, banning alcohol advertising. As study participants noted, tackling the harmful use of alcohol was complex and required robust policies:

“W*e normally speak about substance abuse in three tiers[:] … Demand Reduction [that involves] education …, Supply Reduction [that involves the] South African police service [,] the department of justice [and the] South African Breweries …, and Harm Reduction [that is related to treatment and involves] the department of Health [and] the department of social department”* (Study participant _1).

Substance abuse, including *Nyaope or wunga* (a street drug that has been widespread in South Africa from 2010), was associated with depression and crime, while banning advertising of alcohol was seen as a way of changing the image of alcohol (binge drinking and alcoholism) from being glamourous to categorising it as an illness:

“W*hen [a study of 14 drugs] compared harm to the user [and] … harm to others, alcohol was actually number 1. So the harm to others is actually worse than … cocaine, crack cocaine and crystal meth*” (Study participant _42).

Alcohol control policies tend to focus more on the regulation of alcohol production and distribution. Consequently, priority is given to addressing trade and industry concerns rather than public health issues [7]. Nevertheless, policy formulation and implementation has been more successful in tobacco smoking.

#### Tobacco control policy

Despite the growing opposition from multinational tobacco companies against anti-smoking regulations especially in developing countries [30], the DOH has led the way in controlling tobacco consumption. The Tobacco Products Control Act 21 (1993) that was passed prior to the ending of apartheid has since been amended several times (Table 2). There had been very little or no implementation before 1994 because the apartheid government was keen to protect Afrikaner business interests. It was not until after 1994 that:

“*A new Health Minister Dr Nkosazana Zuma … was prepared to do things that were [in line with] international best practice*” (Study participant _40).

The main drivers of this change of policy were the Left and the Tobacco Action Group (the Heart Foundation, The Cancer Association of South Africa (CANSA) and the Council against Smoking). Through the Amendment of the Tobacco Products Control Act (1999) that was passed after the 1997 ANC conference, advertisements of tobacco products and tobacco smoking in public buildings were banned. The Act also provided for the allocation of smoke-free zones [7]. This was not without opposition from the tobacco industry, the media, the Democratic Alliance (DA) and (surprisingly) the Pan African Congress (PAC):

“*The industry obviously did not want the legislation at all and they opposed everything and anything the government said … the SABC [South African Broadcasting Corporation] was worried about loss of advertising and revenues. We had … big media houses going to parliament and saying [that] if you ban tobacco advertising…they will close down. Then … the Freedom of Expression Institute opposed the legislation … but the courts found that the ban on advertising was constitutional”* (Study participant _40).

The Tobacco Action Group responded to claims about potential job losses, harm to the economy and freedom of speech:

*“Journalists were even telling us that … pro-legislation [accounts] would not be published by the newspaper editors because their own interests were different and [they] policed every story we told … One of the concerns was to make sure that [there was protection of] the main victims of secondary smoking … [that is,] women … [Moreover,] advertising … [was] banned to protect everybody but particularly … to make sure that younger women didn’t think smoking was clever, smart and glamorous … [the] tobacco industry was targeting younger women with their advertising”* (Study participant _40).

Some organisations argued that they were excluded in policy formulation, and that regulations and/or tax increases would raise their costs, reduce their market share and reduce their profits. Notwithstanding these objections, the regulations resulted in the reduction in tobacco smoking by 22% between 1999 and 2009, especially among the youth [5, 10]. Nevertheless, South Africa still has one of the highest smoking rates in Africa [5, 10]. Although the policies passed since 2000 changed practice, continuing problems with smoking have compelled the DOH to “revise[e] its regulations to enforce plain packaging and clean air regulations, regulate e-cigarettes, and increase taxes to revitalise efforts to reduce tobacco use” ([5], p. 1). The government resolved that, for a healthy nation, tobacco controls should be accompanied by tackling the lack of physical activity and unhealthy diets.

#### Policy on physical inactivity

To this end, in 2009 the ANC committed to supporting the promotion of healthy lifestyles. While the private sector has often concentrated on cost-analysis of Prescribed Minimum Benefits (PMB) concerning lifestyle “choices” by individuals as regulated by the Medical Schemes Act 1998 (Study parcipant_32), study participants concurred that healthy living should involve some form of exercise. However, participants argued that physical inactivity resulted from factors such as inadequate education and infrastructure, and the lack of bicycle and walking lanes:

“T*oo many people are getting up in the morning, getting into their car or into a taxi right outside their house … if you want people to ride bicycles you have to create the [safe] environment [including policing] for people to ride bicycles. You try and ride a bicycle in Pretoria, you gonna get knocked over … [what is required are] physical planners [who ensure that the building of roads is accompanied by] lane[s] for bicycles [and walking]*” (Study participant _9).

Programmes introduced to tackle physical inactivity that is a major risk factor for NCDs, include:

“*Sport and recreation … to address (hypertension, high blood pressure) NCDs in elderly people … the Golden games [have subsequently been ‘appropriated’ by] the National Department of Social Development*” (Study participant _44).

#### Policy on unhealthy diets

Alongside the promotion of physical activity are policies that target unhealthy diets, including salt reduction (2013) and trans-fats (2011) in processed foods:

*“We believe that if you teach a person what and how to eat, they will hold onto that till [they] grow old, and old grannies will continue to teach the next generation as well”* (Study participant _14).

By 2011, the Minister for Health began to prepare the country for the salt reduction regulations, arguing that “reducing salt intake in just bread only would save close to 6500 lives per annum” ([20], p. 2). By 2013, the Salt Reduction Regulations were adopted as an intervention for tackling hypertension [7, 31]. Too much salt intake is associated with hypertension, and “*in South Africa hypertension is [also] - - - the major cause of kidney disease*” (Study participant_4). Given that legislation is in place, it remains to be seen how successful the implementation will be:

“*The companies [had] until 2016 for the first target [for reducing salt] and then to 2019 for the second target … if you do it gradually, then people don’t even know they are tasting anything different and they get used to it*” (Study participant _9).

In 2016, the national treasury drew up proposals for the taxation of sugar-sweetened beverages [32, 33]. The proposals for taxing sugar-sweetened beverages were not only debated by the national treasury and DOH, but stakeholders from civil society organisations, industry, research and academics also participated in the drafting and refinement of the taxation regime [7, 34].

High sugar consumption is associated with obesity and diabetes. The South African National Health and Nutrition Survey (SANHANES) that involved more than 25,000 participants reported that there were significantly more females who were overweight and obese (39.2 and 24.8%, respectively) than males (20.1 and 10.6% respectively) [35]. The situation is so serious that South Africa is now considered “the fattest nation in Africa” [35]:

“W*e need to explain to the public that even though they don’t feel sick, they are sick. They are seriously obese and they are on the brink of developing diabetes, they have got high blood pressure but they don’t feel sick yet … Also educate teachers in high schools … [and make children aware] of the long-term risks of being physically inactive … [and incorporate healthy lifestyles into] the curriculum in schools*” (Study participant _18).

The taxation of sugar-sweetened drinks aims to “cut the number of obese people by 220,000 in 3 years” [4]. With this in mind, the Minister for Finance (2017) assured the country that the sugar tax would be implemented [36]. However, funds for implementing large-scale programmes to prevent and control NCDs are inadequate.

### Challenges in implementing NCD policies

#### Funding priorities focusing on infectious diseases

Despite the existence of policies, NCD prevalence seems to be increasing rather than decreasing. Specifically, “the number of deaths due to NCDs … [was] similar [in 2010] to the number from HIV/AIDS and tuberculosis (TB) combined” [4]. Yet “international funders continue to focus on HIV/AIDS” [4]:

*“[Funding is concentrated on] T.B., malaria, HIV … there is a problem with prioritizing disease, [particularly] if you look at kidney disease per se”* (Study participant_4).

To ensure the success of NCD policies, funding must also be linked with community participation *vis-a-vis* multi-sectoral action:

“*If the government can give out a million for a few seconds advert on TV, the government can give out money for full studies that are community based, that involve people to change … We have to include community involvement … even the communication strategy needs to focus on the people*” (Study participant _31).

While emphasis was placed on people owning the policies, there was also concern that policies were not well co-ordinated.

#### Lack of multi-sectoral action

Controlling NCDs is not helped by the tendency of departments and organisations to work in silos, focusing only on specific NCDs without necessarily viewing policy formulation in a holistic way:

“*[Prevention of NCDs] is more at a company level. For instance…TB is an occupational disease … we support companies but with NCDs - - - we just leave it to the company*” (Study participant _26).

In addition, multi-sectoral action can only be successful if data problems are addressed.

#### Lack of data

Concerns relating to the unavailability of panel data were cited:

“*The baseline data that is used to set the target are sometimes questionable because of data collection. We get our information from the district health information system DHIS that is how the district health data is collected … from [the] clinic level to national level. There is a lot of data problems in that system due to incorrect data and so on*” (Study participant _12).

Meeting the NCD reduction targets requires consistent and comparable data to identify patterns and trends, and thus inform policy on how NCD risk factors can be monitored and controlled. Non-disclosure of data and non-cooperation of stakeholders in the implementation process poses problems to policy.

#### Interference from industry

The Control of Marketing Alcohol Beverages Bill (2013) is a case in point. Despite the potential for reducing road fatalities and minimising exposure of alcohol to minors, the alcohol industry has been opposed to the enactment of strict controls. Big business and the media stifled debate on the Bill and used potential job losses to argue against the banning of alcohol advertising. On warning labels:

“*They [the industry] said to us, alright take us to court … one of their high people [did] admit that they had been duping us … they were prepared to go to court but at the same time saying that warning labels have not [had] impact on drinking. This is what they say, so I ask if there is absolutely no impact why so scared?*” (Study participant _9).

At a time of growing youth unemployment, such threats were sufficient to put pressure on the government to withdraw the Bill. Delaying tactics involved asking the government to embark on further research on the impact of alcohol advertising:

“*a request for more research and we also know that most of it is because the alcohol industry is gonna do whatever it can to ensure that there’s a delay in the public discussions on this and the implementation of the alcohol [advertising] ban. They see that of all the policies … as the biggest threat to the industry*” (Study participant _42).

On a different but related matter concerning the government’s efforts to limit sodium levels, Hoffman and Lee observed that: “industry’s opposition to government intervention lay not only in the political debate of the encroaching powers of a ‘nanny state’ government, but also in the practicality of the proposed measures” ([37], p. 8). The food industry tried different tactics to resist and circumvent the regulations:

“*Things like bread, they said this is the limit, after this our bread is going to collapse … We do not actually believe that it’s not possible for them to find something else at that point. So we have given them a challenge … [and said to them that] we have given you a long time to work this out and it got very complicated because in the United Kingdom they managed to bake bread at lower levels [of salt]. They said wheat is different … There is sort of international food safety regulation. It does not include things like salt. We said, of course it must include things like salt; it makes your food unsafe. So, there’s a whole shift that needs to take place and it is taking place”* (Study participant _9).

### Multi-sectorial action

Given these challenges to policy implementation, it is paradoxical that multi-sectoral action should be so entrenched in policy-making in South Africa. According to Chapter 4 of the Constitution, public participation is a requirement for the development of policy. Bills must be availed to the public for comment before approval by Cabinet. Policies, guidelines and programmes must show evidence (a list of stakeholders) of formulation in a consultative manner. Tobacco Control policies and other NCD policies like reduction of sodium regulations were formulated with the participation of diverse stakeholders (multi-sectoral action) [7]. However, this has not resulted in the use of MSA in implementing NCD prevention and control programmes, the exception being programmes targeting physical inactivity.

## Discussion

Various studies have analysed the nuances and dynamics in the formulation of policies targeting specific NCD risk factors, such as tobacco smoking, harmful use of alcohol and high salt content in processed foods [4, 11, 38]. However, few studies have explored the evolution of NCD prevention policies in South Africa. Not only does this paper trace the evolution of NCD policies, but it also contextualises factors relating to process and the ideological rationale that underpinned the design of policies.

South Africa has several comprehensive policies and programmes that target all the four major NCD risk factors. The policies were formulated long before the global drive for prevention and control of NCDs. They subsequently evolved in tandem with international developments. What was particularly critical in the South African case was the enabling post-apartheid political environment for the formulation of NCD policies. Equity became central to policy-making.

By contrast, since the 1970s, the apartheid regime’s Nationalist Party was closely associated with and funded by the tobacco industry. The regime resisted to pass anti-tobacco legislation. Public health risks caused by tobacco smoking were either minimised or dismissed. For those outside the Nationalist Party circles, illnesses from tobacco smoking were often blamed on the nefarious apartheid regime.

While tobacco smoking in African countries that were not under apartheid also persisted, the failure of governments to enact anti-tobacco legislation was not generally associated with collaboration between ruling political parties and the tobacco industry [39, 40]. In some cases such as Cameroun and Malawi, the importance of tobacco as an important cash crop and source of revenue has complicated policy formulation [40, 41].

In South Africa, on the other hand, the ANC’s political ideology served to shift the trajectory of health policies, systems, and how they function. There are three ideological perspectives that are critical to the understanding of health policies – conservative, liberal and radical [42]. The conservative approach is based on the notion of “equality before the law” ([42], p. 4). From the conservative perspective, the purpose of state intervention in health is to ensure that the law is upheld. The underlying rationale is that actual provision and prices of healthcare should be allocated by the market.

The liberal approach to health care is based on the ideal of “equality of chances” ([42], p. 4). In this regard, state intervention is acceptable to the extent that it helps to improve the health status of the population. What sets the radical approach apart is its underlying rationale that emphasises the “equality of results” ([42], p. 4). From this perspective, state intervention is required to achieve the desired health outcomes. The radical approach in healthcare policy and implementation requires centralized planning and the allocation of resources in achieving the desired health outcomes.

Under apartheid, policies were aligned with a racist ideology that promoted racial exclusion. State intervention was for the protection of the healthcare of a privileged minority. By contrast, the post-apartheid government - a tripartite alliance of the ANC, the South African Communist Party (SACP) and Congress of Trade Unions (COSATU) – sought to redress past inequalities resulting from exclusion and redistribute resources [7]. The policy approach taken by the government since 1994 is reflective of the debates on political ideology and tensions within the tripartite alliance, resulting in a mix of liberal and radical approaches [19].

In terms of NCDs prevention and control, state intervention has tended to follow this mixed approach for the attainment of “health for all”. The ideological stance of the Left (SACP, COSATU, the left-wing of the ANC and the Tobacco Action Group) inevitably influenced the formulation and implementation of tobacco control that ran counter to the anti-regulation position of tobacco multinational companies [30, 42]. However, state involvement in the implementation of other NCD policies such as salt regulation has taken a more liberal approach. The latter is similar to the Bhutan case where there is still a “need to consider policy socio-political and economic factors” [42] in the context of a radical approach.

The purpose of formulating NCD policies is to effect behavioural change and the reduction of NCDs in general. NCD policies are in place, but the prevalence of NCDs has increased except in the case of tobacco smoking. This is not unique to South Africa; rather, it is a global phenomenon particularly in low-income countries [5, 6, 9, 11]. Physical inactivity is particularly a challenge among women in low-income countries and South Africa, in particular. The lack of green spaces for walking in the sprawling urban informal settlements, as well as crime and gender-based violence in South Africa are deterrents to physical activity [43].

### Political influence

At the global level, the political commitment to tackle NCDs also influenced developments on NCD prevention and control in South Africa. In 2011, the United Nations endorsed the political declaration for the control and prevention of NCDs at a meeting in Moscow, Russia [11]. South Africa was a signatory to the United Nations Political declaration for NCDs. In 2012, the World Health Assembly went further to set targets for the reduction of NCDs by 25% by the year 2025 [9]. Through its political declaration on NCDs, South Africa also made the same commitment in 2012. Much as South Africa influenced the global context in setting the pace for the prevention and control of NCDs particularly in tobacco smoking, her policies were also influenced by global events.

### Multi-sectoral action

As indicated earlier, multi-sectoral action was already rooted in South African policy-making before the global community promoted it [7, 44]. It embodied notions of community/public participation in decision making concerning policies and programmes that impact people’s lives. This paper argues that, although multi-sectoral action was part of NCD policy formulation, this did not translate to implementation, meaning that the risks of NCDs have not been reduced.

## Conclusion

This paper set out to analyse the evolution of NCD policies in post-apartheid South Africa. The underlying ideological rationale of the post-apartheid government’s health equity approach, commitment to reduce health inequities and achieve redistribution, is what set it apart from the apartheid period. Thus, an enabling national political climate and leadership exemplified by the Health Ministers, is critical to design of policies for the prevention and control of NCDs.

The contribution of this paper lies in identifying public participation as vital to NCD policy formulation. Implementation is critical to the reduction NCDs, hence the recommendation to integrate multi-sectoral action in NCD policy implementation.

## Abbreviations

AIDS: Acquired immune deficiency syndrome
ANC: African National Congress
ANPPA: An analysis of (NCD) prevention policies in Africa
ARV: Antiretroviral therapy
CANSA: Cancer Association of South Africa
COSATU: Congress of Trade Unions
DA: Democratic Alliance
DHIS: District Health Information System
DOH: Department of Health
GEAR: Growth and Employment Policy
IMC: Inter-Ministerial Committee
MSA: Multisectoral action
NCD: Non communicable diseases
NDP: National Development Plan
NGOs: Non-governmental organizations
PAC: Pan African Congress
PMB: Prescribed Minimum Benefits
SABC: South African Broadcasting Corporation
SACP: South African Communist Party
SANCD: South African Non-Communicable Diseases Alliance
SANHANES: South African National Health and Nutrition Survey
WHO: World Health Organisation
